# Preliminary Report on HIV-1 Vaccine Preparedness in Nigeria: Advantages of Recruiting University Students

**DOI:** 10.3390/v2010073

**Published:** 2010-01-11

**Authors:** Abigail Edubio, Simon Agwale, Marc Bulterys, Dadik Jelpe, John Idoko, Chris Isichei, Ruth Guyit, Alash’le Abimiku

**Affiliations:** 1 Laboratory of Leishmaniasis and AIDS, Department of Zoology, University of Jos, P.M.B 2084, Jos, Plateau State, Nigeria; E-Mails: abigail.edubio@charite.de (A.E.); sagwale@innovativebiotechng.com (S.A.); 2 School of Medicine, University of New Mexico, Albuquerque, New Mexico, USA; E-Mail: bulterys@cn.cdc.gov (M.B.); 3 Plateau State Human Virology Research Center (PLASVIREC), Plateau State Specialist Hospital, Jos, Nigeria; E-Mail: JelpeT@ng.cdc.gov (D.J.); 4 Faculty of Medical Sciences, University of Jos, P.M.B 2084 Jos, Plateau State, Nigeria; E-Mail: jonidoko@yahoo.com (J.I.); 5 Department of Chemical Pathoglogy, University of Jos, P.M.B 2084 Jos, Plateau State, Nigeria; E-Mail: cogom@hisen.org (C.I.); 6 Institute of Education, University of Jos, P.M.B 2084 Jos, Plateau State, Nigeria; E-Mail: ruthieguyit@yahoo.co.uk (R.G.); 7 Division of Epidemiology and Prevention, Institute of Human Virology, School of Medicine, University of Maryland, Baltimore, USA

**Keywords:** Nigeria, HIV, vaccine, recruitment, strategies

## Abstract

The national HIV seroprevalence in Nigeria has risen steeply from about 3% in 1993 to 5–8% in 2001 and now stands at 4.4%. HIV epidemic continues to be a serious threat to the most populous country in Africa with a population of 140 million, with limited use of antiviral drugs that is taken for life since it only suppresses the virus without completely eliminating the virus or leading to cure. Only a change in social behavior and an affordable vaccine can halt the epidemic in Africa. We report here results of a pilot study on the recruitment strategies, sociodemographic aspects and HIV risk behavior of a cohort of normal volunteers recruited at the University of Jos, Nigeria. Our study recorded a high degree of interest and zeal to participate in HIV vaccine studies by volunteers, and demonstrated the superiority of snowballing over invitation by mail, as a recruitment strategy. A cohort of university students may be particularly suitable for conducting HIV vaccine trials because of the assurance of prospective follow-up for up to four years (time to graduation), and a good understanding of the risks and benefits of participation as outlined in the informed consent. We had 100% retention during a follow-up period of two years. Most importantly, the cohort reflected a relatively low HIV seroprevalence, which gives preventive programs the potential to blunt or halt the epidemic.

## 1. Introduction

The HIV epidemic in sub-Saharan Africa is confounded by the complexity of the virus [1] and the unaffordability of current antiretroviral drugs. Only a safe, effective and affordable vaccine, along with health education and behavioral changes, can halt the rapid spread of the virus. Multiple HIV vaccine efficacy trials running in parallel will be necessary in different geographic areas, while paying close attention to HIV subtypes circulating locally [2–6]. A critical challenge will be to recruit, counsel and retain a sufficient number of vaccine trial volunteers and to ensure community involvement in all aspects of HIV vaccine trial development efforts in Africa [7]. We report here results of a pilot study on the recruitment strategies, sociodemographic aspects and HIV risk behavior of a cohort of HIV vaccine trial volunteers recruited at the University of Jos, Plateau State, Nigeria.

## 2. Methods

During the two academic years, 231 volunteers were screened for the potential inclusion into a phase I/II HIV vaccine trial cohort. All candidates irrespective of HIV status received pre- and post-test counseling and detailed information on the study objectives and procedures by trained research personnel. A detailed questionnaire was administered and each volunteer received a confidential 3-digit number which identified them henceforth. All recruited volunteers were undergraduate students with at least three years of study remaining at the university. A blood specimen was drawn for HIV antibody screening by Elisa (Vironostica, Organon Teknika, Benelux) followed by Western blot confirmation at the Plateau State Human Virology Research Center (PLASVIREC). Whole blood from each volunteer was dotted on filter paper and stored at −20 °C for future molecular analysis. Students with HIV-negative serology consented to be enrolled and continue to be followed in a prospective cohort to hypothetically test the safety and immunogenicity of a promising HIV-1 vaccine candidate in a Phase I clinical trial. Students were either contacted by their peers or invited by mail to participate. One of the study objectives was also to determine which recruitment method would be most successful in recruiting university students into an HIV vaccine trial.

## 3. Results

Two hundred and thirty-one volunteers were screened. Nine of the 231 (3.8%) were HIV-1 positive, which is comparable to a national seroprevalence of 4.4% [8]. Out of the 222 that were HIV-1 seronegative, 200 were recruited into the study and followed for over two years. Table 1 summarizes the sociodemographic characteristics of the cohort during two years follow-up. All eight departments of the university and 44 of Nigeria’s 360 Nigerian ethnic groups were represented. The median age was 21 years; 63 percent of volunteers were Christians and 19 percent were Moslems. Jos is situated in the north central part of Nigeria which is predominantly a Christian community. Nineteen percent were married while the majority (81%) was single. Condom use was rare and 82 volunteers (35.5%) reported more than one concurrent sexual partner. Snowball recruitment through friendship networks yielded far more volunteers (63%) than direct contact by invitation letter sent in the mail (37%). Students were motivated by access to free information on HIV/AIDS, the urgent need for an effective vaccine, and the opportunity to participate in an AIDS awareness and control program. As an incentive, the provision of free key holders emblazoned with a condom and a safe-sex message was also highly successful.

## 4. Discussion

The seroprevalence seen among young university students in Jos reflects the HIV epidemic observed in other parts of the country with a national estimate of 4.4% prevalence [8]. Much higher seroprevalence rates have been reported in past national sentinel survey and among high risk groups in Plateau State and other parts of the country [3,8–9]. Preliminary results from testing of students with high risk behavior who attend the university clinic show a seroprevalence of 23% (unpublished data); thereby making it possible to use modified recruitment techniques to specifically recruit volunteers with high risk practices for a phaseII/III HIV vaccine trial within this same population of over 6000 students. In developing countries where the level of education is relatively low, a cohort of university students may be particularly suitable for conducting HIV vaccine trials because of the a good understanding of the risks and benefits of participation as outlined in the informed consent form, the success of snowball enrollment, the enthusiasm among university students in participating in a vaccine trial, and a relative ease and assurance of prospective follow-up for up to four years (time to graduation). As was shown previously in Rwanda [10], the low prevalence of HIV among young students could mask an emerging high HIV incidence in this population, reflecting concurrent high-risk sexual behavior. We found that a relatively significant proportion (35.5%) of students in our cohort had more than one concurrent sexual partner. Nevertheless, the low prevalence gives educators a window of opportunity for preventive programs aimed at increasing skills to prevent HIV infection among young students.

## Figures and Tables

**Table 1.** Socio and demographic characteristics of a cohort of normal volunteers from the University of Jos, Nigeria.

**Cohort characteristics**	**No.**	**Percentage (%)**

Total number of volunteers	231	-

HIV-1 seropositivity of volunteers at recruitment	9	3.8

Total number recruited and enrolled	200	

Gender:		
• Females	67	29
• Males	164	71

Recruitment strategy:		
• invitation letter	85	36.7
• snowballing	145	62.7

Religious denomination of volunteers:		
• Christians	145	62.7
• Moslems	44	19
• Others	42	18

High risk behavior:		
• Having multiple sex partners	82	35.5
• Regular use of condoms	Rare	-

Retention and compliance:		
• number followed up for 2 years	200	100
• number that complied with study protocol	200	100
